# Molecular Framework of Mouse Endothelial Cell Dysfunction during Inflammation: A Proteomics Approach

**DOI:** 10.3390/ijms23158399

**Published:** 2022-07-29

**Authors:** Michael T. Rossi, Jordan C. Langston, Narender Singh, Carmen Merali, Qingliang Yang, Salim Merali, Balabhaskar Prabhakarpandian, Laurie E. Kilpatrick, Mohammad F. Kiani

**Affiliations:** 1 Biomedical and Data Sciences Division, CFD Research Corporation, Huntsville, AL 35806, USA; mrossi1130@gmail.com (M.T.R.); narender.singh@cfdrc.com (N.S.); prabhakar.pandian@cfdrc.com (B.P.); 2 Department of Bioengineering, Temple University, Philadelphia, PA 19122, USA; jordan.langston@temple.edu; 3 School of Pharmacy, Temple University, Philadelphia, PA 19140, USA; carmen.merali@temple.edu (C.M.); salim.merali@temple.edu (S.M.); 4 Department of Mechanical Engineering, Temple University, Philadelphia, PA 19122, USA; qingliang.yang@temple.edu; 5 Center for Inflammation and Lung Research, Department of Microbiology, Immunology and Inflammation, Lewis Katz School of Medicine, Temple University, Philadelphia, PA 19140, USA; laurie.kilpatrick@temple.edu

**Keywords:** inflammation, endothelium, proteomics, organ heterogeneity

## Abstract

A key aspect of cytokine-induced changes as observed in sepsis is the dysregulated activation of endothelial cells (ECs), initiating a cascade of inflammatory signaling leading to leukocyte adhesion/migration and organ damage. The therapeutic targeting of ECs has been hampered by concerns regarding organ-specific EC heterogeneity and their response to inflammation. Using in vitro and in silico analysis, we present a comprehensive analysis of the proteomic changes in mouse lung, liver and kidney ECs following exposure to a clinically relevant cocktail of proinflammatory cytokines. Mouse lung, liver and kidney ECs were incubated with TNF-α/IL-1β/IFN-γ for 4 or 24 h to model the cytokine-induced changes. Quantitative label-free global proteomics and bioinformatic analysis performed on the ECs provide a molecular framework for the EC response to inflammatory stimuli over time and organ-specific differences. Gene Ontology and PANTHER analysis suggest why some organs are more susceptible to inflammation early on, and show that, as inflammation progresses, some protein expression patterns become more uniform while additional organ-specific proteins are expressed. These findings provide an in-depth understanding of the molecular changes involved in the EC response to inflammation and can support the development of drugs targeting ECs within different organs. Data are available via ProteomeXchange (identifier PXD031804).

## 1. Introduction

Inflammation, in response to infection or injury, activates the vascular endothelium, resulting in pro-inflammatory signaling, coagulation, increased barrier permeability and excessive leukocyte trafficking to vital organs, such as the lungs, liver and kidneys [1,2,3,4]. This increased leukocyte trafficking is associated with tissue damage, multiple organ dysfunction and increased mortality. Thus, the endothelium has a critical role in the cytokine-induced changes and is an important therapeutic target [5]. While the endothelial cells (EC/ECs) share multiple common properties, EC heterogeneity results in organ-specific variations in the EC structure, function and mechanisms regulating leukocyte trafficking into key organs [6,7,8]. Sepsis, for example, is an important inflammatory disease which is a major health issue in the United States, with over 1.7 million cases/year and more than 270,000 deaths/year, despite appropriate antibiotic therapies [9,10]. In sepsis, patients often die of organ failure, and vascular EC barrier function plays a critical role in the early course of organ damage [11,12]. Thus, it is important to understand EC heterogeneity and its impact on cytokine-induced changes as observed in sepsis.

In order to develop appropriate therapeutics targeting the vascular endothelium, studies are needed to identify organ-specific EC responses to inflammatory signals. While several studies have analyzed the functional changes in the ECs of different organs during cytokine-induced changes as observed in sepsis [1], the underlying molecular mechanisms of this differential response have not been studied. As inflammation is a highly dynamic process, the level of protein alterations may vary during the different stages of disease [13]. Compared with genetic analyses, which often provide indirect clues about cell function, proteomic analysis can give direct insight into the protein expression in organ-specific ECs and help bridge the genotype–phenotype gap [14,15]. Nonetheless, few previous studies have identified the possible protein regulation patterns or evaluated the interaction and differential expression among various proteins in organs, such as the lungs, liver and kidneys during inflammation [16,17]. Specifically, most previous proteomics studies have focused on identifying diagnostic biomarkers for cytokine-induced changes [18,19], or have used genomics or transcriptomics to group patients into different endotypes [20,21,22], rather than developing a comprehensive, mechanistic understanding of the disease and the role of EC heterogeneity in tissue damage and organ failure.

In this study, we use a combination of in vitro and in silico approaches to present a comprehensive analysis of the dynamics of proteomic changes in mouse lung, liver and kidney ECs, following exposure to a cocktail of clinically relevant proinflammatory cytokines (cytomix: TNF-α, IL-1β and IFN-γ) [23,24,25,26]. We hypothesize that during the inflammatory process, the organ-specific EC proteomic expression changes over time, and there are unique EC-specific proteins that are differentially expressed.

## 2. Results

### 2.1. Global Proteomic Analysis Identifies Differentially Expressed Proteins in ECs in Response to Inflammatory Stimuli

**Figure 1:**
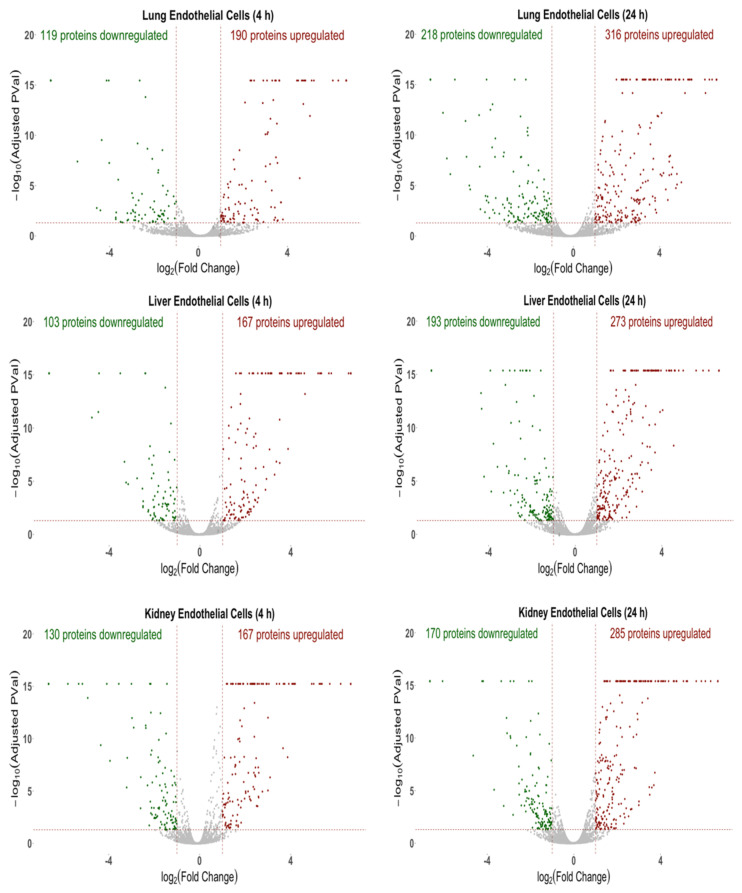
Volcano plots of the protein changes for the 4 h and 24 h cytomix-treated ECs as compared to control. For all three organs, the red represents upregulated proteins, green represents downregulated proteins and gray represents proteins that were not significantly altered in response to cytomix treatment.

The global proteomic analysis, comprising of more than 6000 proteins, identified both unique and common proteins between the ECs specific to the lung, liver and kidney. Figure 1 highlights the volcano plots for the upregulated, downregulated and insignificantly changed proteins between the control (buffer-treated) and cytomix-treated ECs of each organ at 4 h and 24 h. Each dot represents the fold change (log2 fold) versus the significance (−log10 (*p*-value)). The red dots represent the upregulated proteins, the green dots represent the downregulated proteins and the gray dots represent the proteins that were not significantly altered in response to the cytomix treatment. At the 4 h timepoint, the cytomix-treated lung ECs had 190 proteins upregulated and 119 proteins downregulated, as compared to the control ECs. The liver ECs had 167 proteins upregulated and 103 proteins downregulated, and the kidney ECs had 167 proteins upregulated and 130 proteins downregulated in response to the cytomix treatment. At 24 h post cytomix treatment, there were further significant alterations in the protein expression in lung ECs, resulting in 316 upregulated proteins and 218 downregulated proteins, as compared to the control ECs. Similarly, the liver ECs had 273 upregulated proteins and 193 downregulated proteins, while the kidney ECs had 285 upregulated and 170 downregulated proteins, as compared to the control ECs. The upregulated and downregulated protein lists in each of the organs at 4 h and 24 h are listed in the “4 h Upregulated Proteins”, “4 h Downregulated Proteins”, “24 h Upregulated Proteins” and “24 h Downregulated Proteins” Excel files in the Supplementary Materials. These differential expression patterns at 4 h and 24 h demonstrate that the cytokine-induced changes as observed in sepsis not only upregulate a significant number of proteins, but also play a major role in the downregulation of the EC proteins.

**Figure 2:**
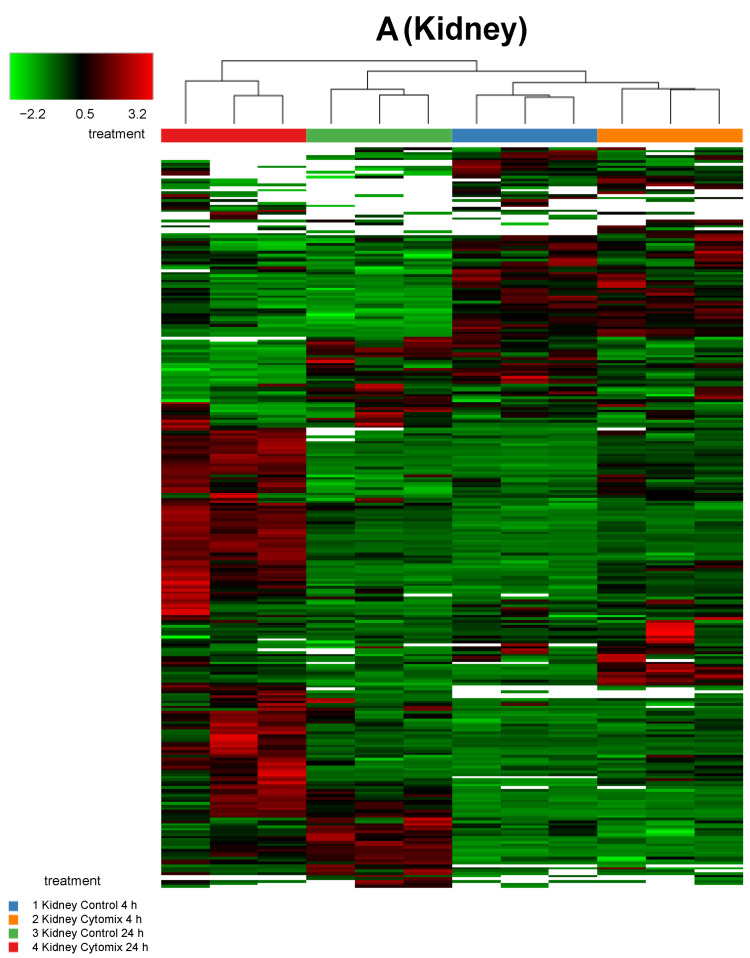

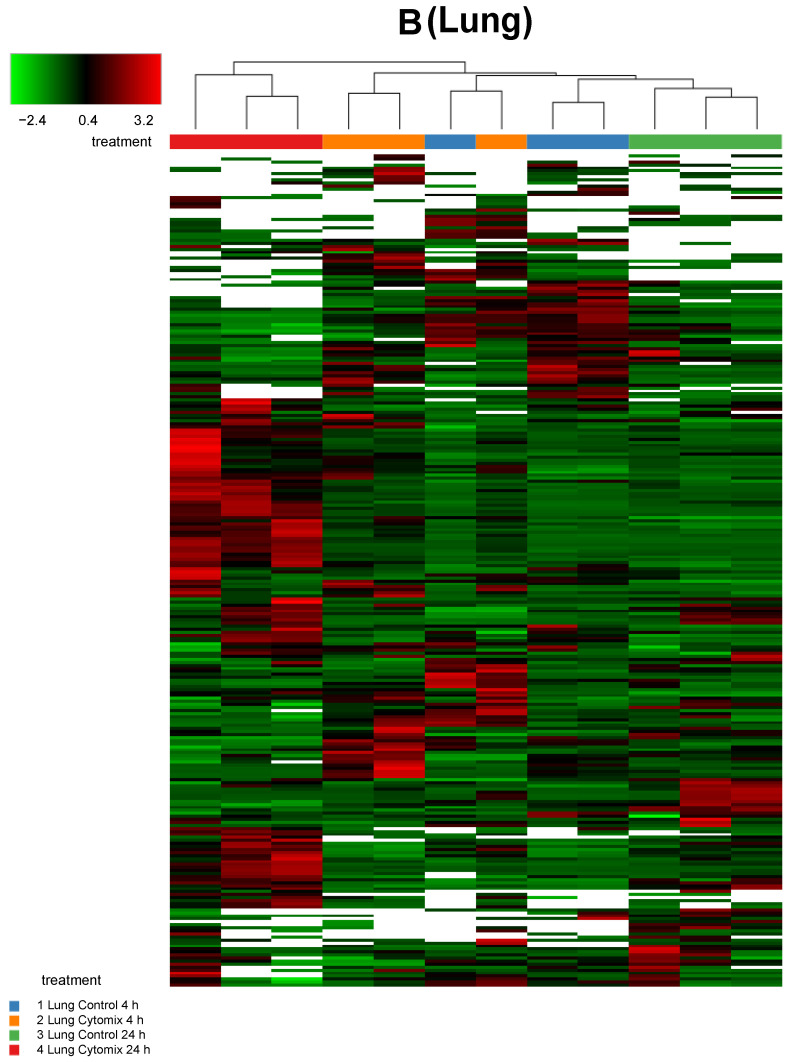

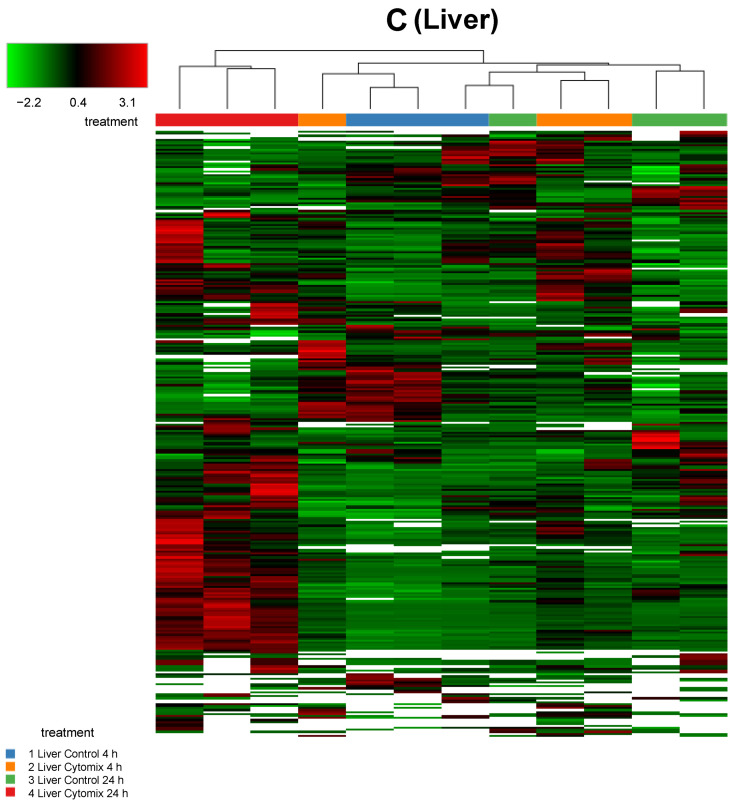
Sample clustering of heatmaps highlighting the overall similarity and differences in normalized protein expression (*p* < 0.05) across control and cytomix conditions for all replicates in the kidney (panel **A**), lung (panel **B**) and liver (panel **C**) at 4 h and 24 h. The white bars in the heatmaps represent those proteins that did not show any expression. The color bars on top of the heatmap represent different treatment groups. Blue bars indicate control at 4 h, orange cytomix at 4 h, green control at 24 h and red cytomix at 24 h. The color key in the top left shows whether protein expression was above or below the mean.

In order to investigate any commonality between the biological replicates across the conditions for an EC phenotype, we generated heatmaps of the proteomic changes. Figure 2 shows the heatmaps of the differentially expressed proteins (*p* < 0.05) for kidney, lung, and liver at 4 h and 24 h normalized protein expression across the control and the cytomix biological replicates. The heatmaps are clustered by sample (or replicate) and thus highlight which samples showed a similar expression for a particular condition across the organs. They provide a qualitative image of the changes occurring in the organ-specific ECs at 4 h and 24 h post cytomix treatment, highlighting the heterogeneity of protein expression between the organs and across time. As shown, in Figure 2, the treatment groups in the kidney ECs cluster together more closely compared to the liver and lung, highlighting that kidney ECs have a greater uniformity between the control or cytomix replicates over time.

**Figure 3:**
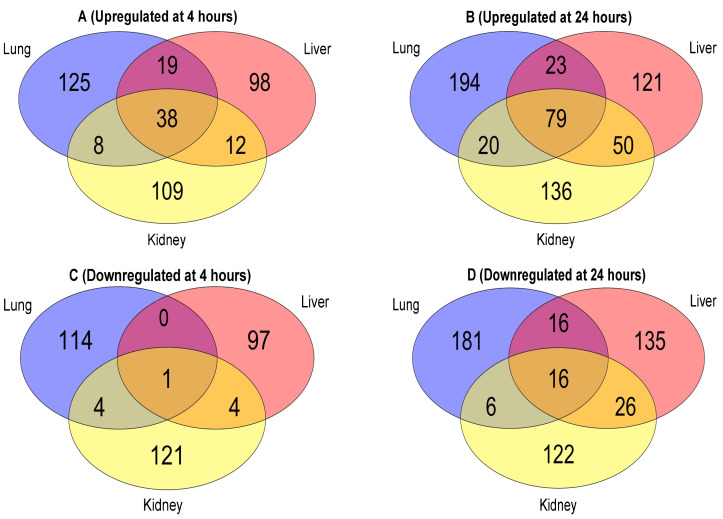
Venn diagrams of the EC upregulated proteins at 4 h (panel **A**); upregulated proteins at 24 h (panel **B**); downregulated proteins at 4 h (panel **C**); and downregulated proteins at 24 h (panel **D**).

As shown in the Venn diagrams, the number of proteins commonly expressed between these organs increases substantially from 4 h (Figure 3A) to 24 h (Figure 3B) after the cytomix treatment. At 4 h post cytomix treatment, 38 of the proteins were shared between all three organs, 12 shared between the kidney and liver, 8 shared between the kidney and lung and 19 shared between the lung and liver. Following 24 h cytomix treatment, 79 proteins were shared between all three EC types, 50 between the kidney and liver, 20 between the kidney and lung and 23 between the lung and liver, highlighting the progression of the cytokine-induced changes over time. The increase in the number of the common proteins between the EC phenotypes indicates more uniformity in the proteomic expression during the progression of inflammation. More importantly, both the liver and kidney ECs showed a minimal increase in the number of the unique proteins at 23 and 27 proteins, respectively, from 4 h to 24 h, while the lung ECs showed an increase of 69 unique proteins at 24 h compared to 4 h. The number of unique and common downregulated proteins between the three organs at 4 h and 24 h are shown in Figure 3C,D, respectively. Following the trend observed with the upregulated proteins, the number of downregulated proteins commonly expressed between all three organs increased from 1 at 4 h (Figure 3C) to 16 at 24 h (Figure 3D), post cytomix treatment. At 4 h post cytomix treatment, four downregulated proteins were shared between the kidney and liver, four between the lung and kidney and no proteins were shared between the lung and liver, highlighting the unique downregulated proteomes in the lung and liver. At 24 h post cytomix treatment, 16 downregulated proteins were shared between the lung and liver, 6 between the lung and kidney and 26 between the liver and kidney, which again suggests that, as inflammation progresses, there is an increase in uniformity in the proteomic expression. Similar to what was observed with the upregulated proteins, while the number of the unique downregulated proteins in the liver and kidney increased at 24 h by 1 and 38, respectively, the number of the unique downregulated proteins in the lung increased by 67. The common and unique protein lists across the organs at 4 h and 24 h are listed in the “Common and Unique Downregulated 4 h Proteins”, “Common and Unique Downregulated 24 h Proteins”, “Common and Unique Upregulated 4 h Proteins” and “Common and Unique Upregulated 24 h Proteins” Excel files in the Supplementary Materials. These findings indicate that there are large numbers of organ-specific changes in protein expression in the ECs over time.

### 2.2. Functional Enrichment Analysis

**Figure 4:**
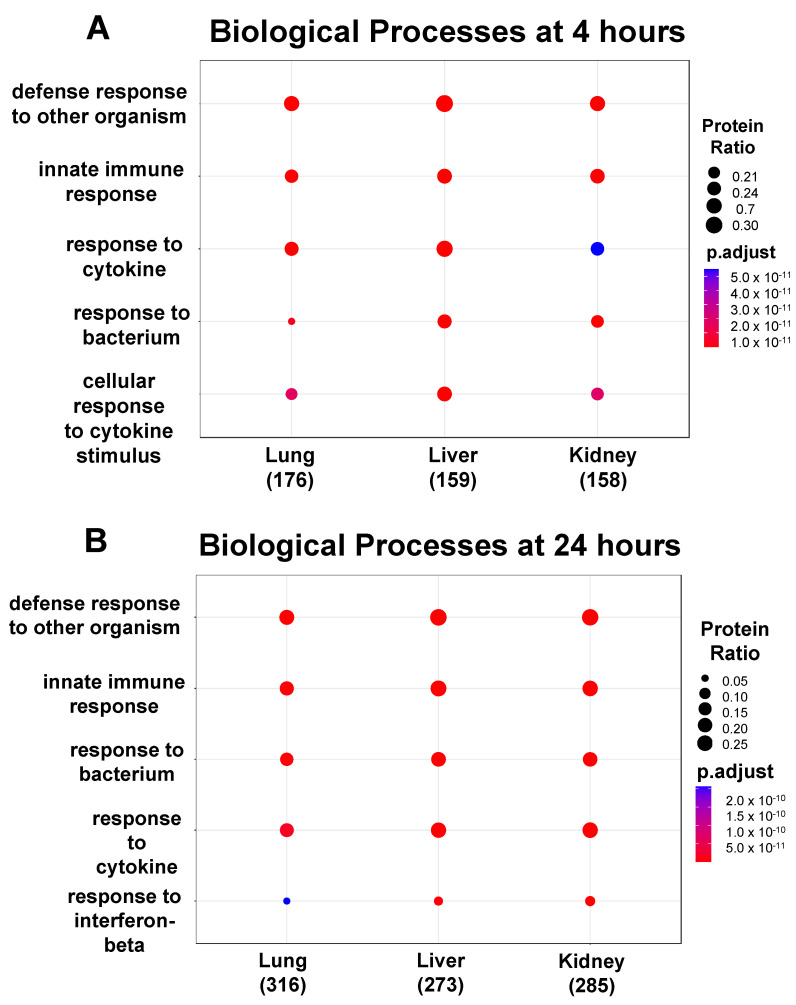

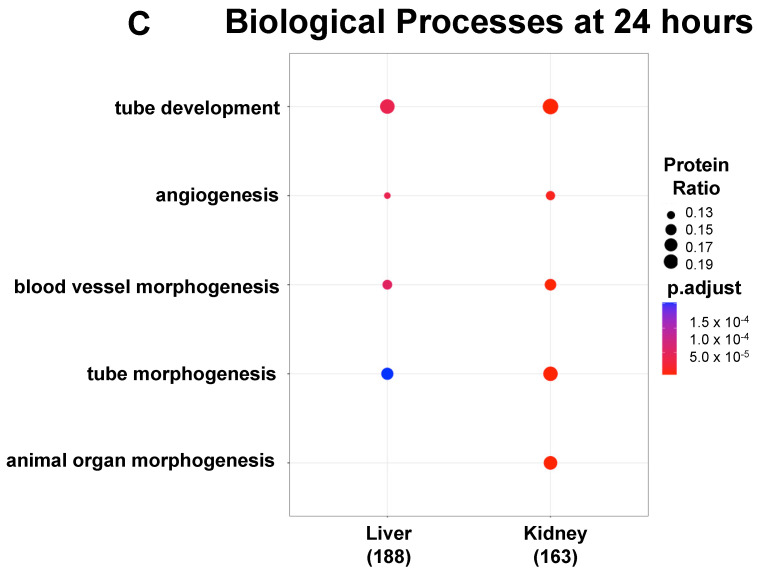
Functional enrichment analysis showing the top 5 GO BPs expressed in the ECs. Over time, 4 of the top 5 GO BPs observed in the upregulated proteins at 4 h (panel **A**) are also present at 24 h (panel **B**). There were no BPs observed in the downregulated proteins at 4 h, but at 24 h (panel **C**), 4 and 5 significant GO BPs were identified in the liver and kidney, respectively.

Based on the proteomic analysis, we then performed functional enrichment analysis by using (a) Gene Ontology (GO) to determine how the different classes of significant (*p* < 0.05) Biological Processes (BPs) regulate EC function during inflammation (summarized in the panels in Figure 4) and (b) the PANTHER database to identify the classes of the upregulated and downregulated proteins common between the three EC phenotypes at 4 h and 24 h (summarized in Table 1, Table 2 and Table 3). GO and PANTHER summarize how the differentially expressed proteins play a significant role in regulating the specific BPs during the cytokine-induced changes in ECs. The BPs can be defined as the biological objective to which a gene or gene product contributes through a series of molecular functions [27]. Thus, the BPs are normally described by the functional role that they play in cells, tissues and organs. As shown in Figure 4, the lung, liver, and kidney ECs shared the same top five GO hits in each BP class at 4 h (panel A) and 24 h (panel B) post cytomix treatment. Furthermore, four of these top five BPs observed at 4 h are still present at 24 h. However, while these BPs similarly activate the immune system, they appear to be doing so in slightly different ways. For example, the liver and kidney ECs appear to have a higher percentage of their proteins involved in the response to the bacterium class when compared to the lung ECs at 4 h (Figure 4, panel A). The response to the bacterium process had 39 proteins upregulated for liver, 32 upregulated for lung and 35 upregulated for kidney. The defense response to the organism had 47 for lung, 49 for liver and 42 for kidney. The response to cytokine had 44 for lung, 46 for liver and 37 for kidney. The innate immune response had 41 for lung, 41 for liver and 40 for kidney. The cellular response to cytokine stimulus had 37 for lung, 41 for liver and 35 for kidney, further highlighting the response of various ECs to inflammation and providing insight into the signaling response. Figure 4, panel B, shows that, while the response at 24 h is similar to that at 4 h, a distinct pattern of uniformity of the proteins across all of the organs is observed. For the proteins, the defense response to the organism had 47 for the lung, 78 for the liver and 79 for the kidney. The innate immune response had 57 for the lung, 69 for the liver and 68 for the kidney. The response to bacterium had 48 for the lung, 56 for the liver and 56 for the kidney. The response to cytokine had 54 for the lung, 62 for the liver and 68 for the kidney. Finally, the response to interferon-beta had 15 for the lung, 16 for the liver and 21 for the kidney. Similar to the 4 h response of the bacterium class, the lung ECs had the lowest percentage of proteins compared to the other BPs for the response to interferon-beta. The “Innate Immune response GO BP”, “Response to Bacterium GO BP”, “Response to Cytokine GO BP”, “Response to Interferon-beta GO BP”, “Cellular Response to Cytokine Stimulus GO BP” and “Defense Response to Other Organism GO BP” Excel files in the Supplementary Materials provide more detailed information on these proteins. Figures S1 and S2 in the Supplementary Materials’ Word file highlight the number of common and unique proteins upregulated between the top five GO BPs at 4 h and 24 h, respectively, across the ECs.

No significant GO BPs were observed in the downregulated proteins either in the lung, liver or kidney at 4 h, or in the lung at 24 h. However, as shown in Figure 4 panel C, four BPs in common in the downregulated proteins at 24 h in the liver and kidney included the BP classes tube development, angiogenesis, blood vessel morphogenesis and tube morphogenesis, suggesting that angiogenesis and blood vessel formation processes are dysregulated during inflammation. More information on the downregulated proteins at 24 h in the liver and kidney can be found in the “Tube Development GO BP”, “Tube Morphogenesis GO BP”, “Angiogenesis GO BP”, “Blood Vessel Morphogenesis GO BP” and “Animal Organ Morphogenesis GO BP” Excel files in the Supplementary Materials. In summary, these data support the concept that the proteomics of the organ-specific ECs evolve from a more heterogeneous to a more uniform response over time during inflammation.

**Figure 5:**
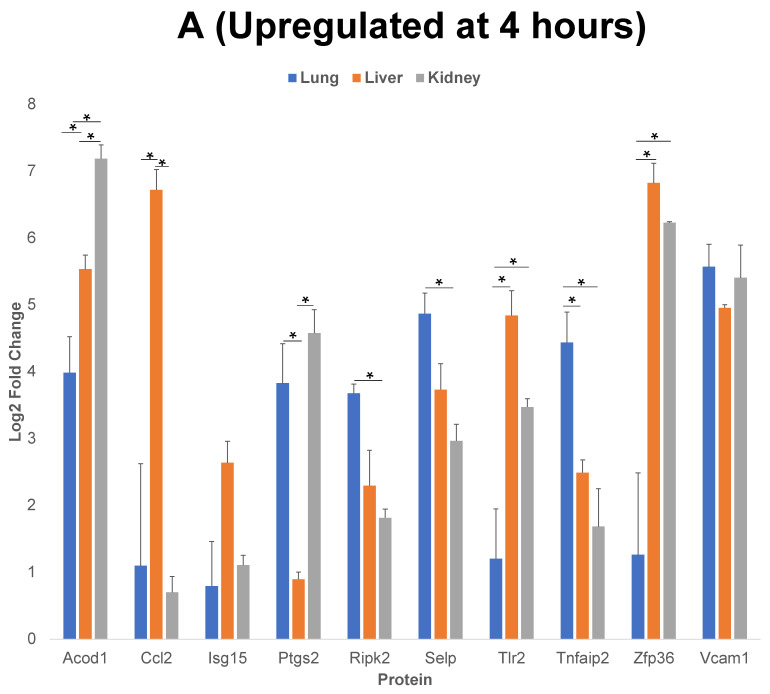

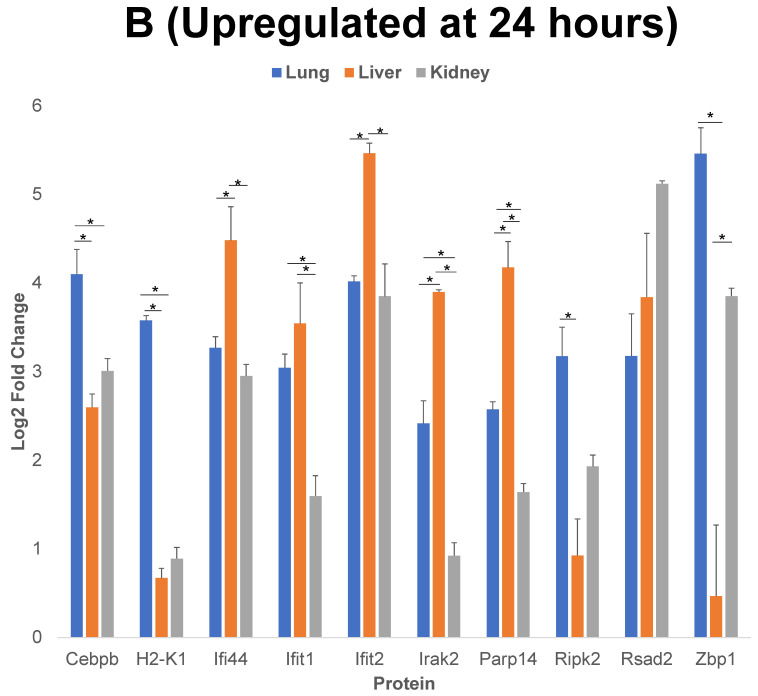

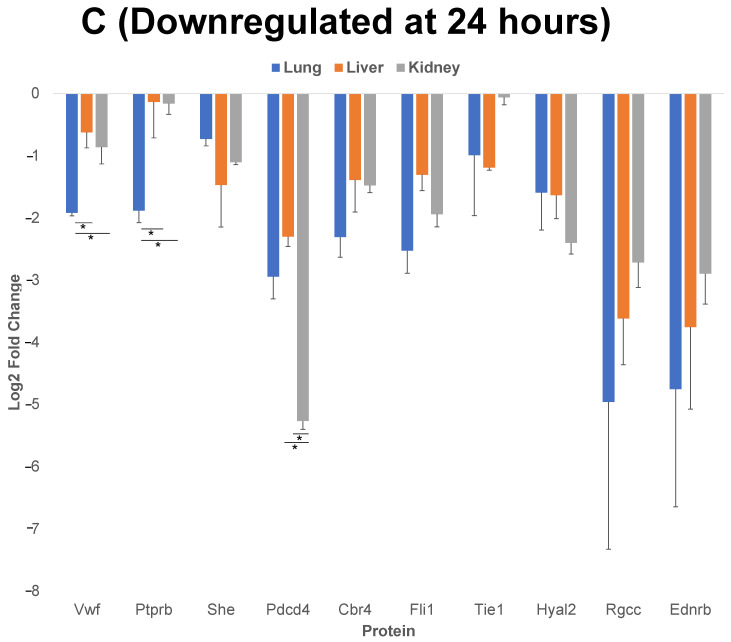
Comparison of 10 of the proteins that have the highest level of upregulation or downregulation compared to control post cytomix treatment at 4 h and 24 h. Panels A and B represent the 4 h and 24 h upregulated proteins, respectively, and panel C represents the downregulated proteins at 24 h. These bar plots highlight the differential expression of proteins across organs. The *y*-axis represents the log2 level change compared to the background levels of each protein. The blue, orange and gray bars represent lung, liver and kidney ECs, respectively. Data are plotted as mean ± SEM (*n* = 3). Analysis of Variance (ANOVA) with Tukey post-hoc test was used to identify statistically significant differences. The “*” symbol indicates that there was a significant difference (*p* < 0.05) between organs.

**Figure 6:**
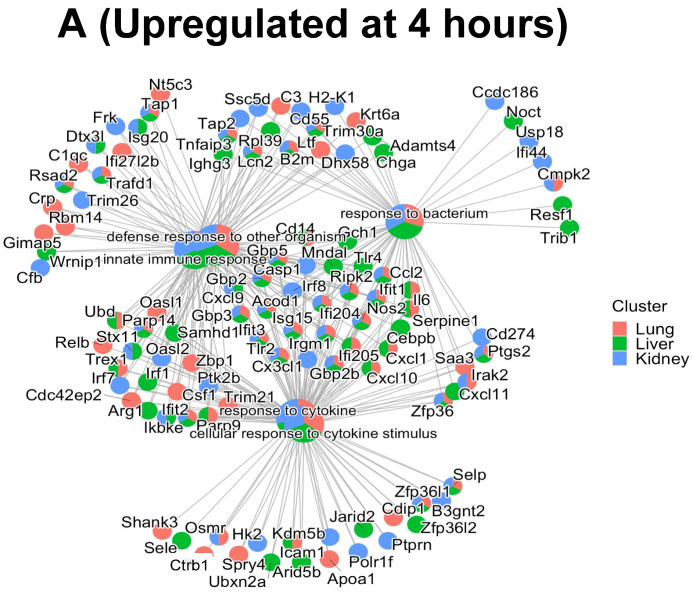

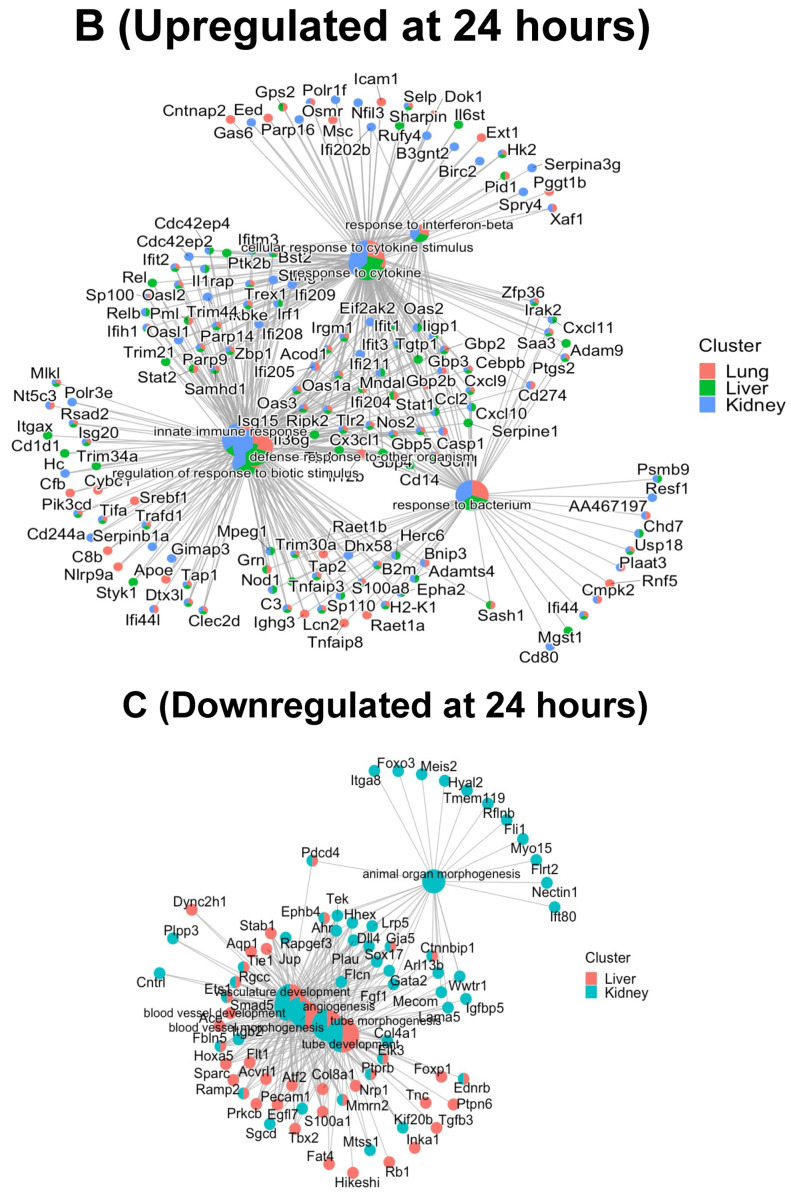
Cnetplots highlighting the interaction of commonly shared proteins between the top 5 GO BPs upregulated at 4 h (panel **A**); 24 h (panel **B**) and downregulated at 24 h (panel **C**) after cytomix treatment.

In this study, we focused on the GO BPs that are relevant to inflammation and common between the three EC phenotypes (as shown in Figure 4, Figure 5 and Figure 6 in the manuscript); however, there were several BPs that were unique to each EC phenotype. For example, in the lung, unique GO BPs include the B-cell-mediated immunity (Nsd2, Il6 and C1qc), immunoglobulin-mediated immune response (Gimap5, Trex1, Nsd2) and regulation of natural killer cell-mediated immunity (Clec2d, Gimap3 and Tap1). In the liver, the unique GO BPs include leukocyte proliferation (Gja1, Cebpb and Tlr4), alpha-beta T-cell activation (Bcl11b, Irf1, Cd44) and EC proliferation (Arg1, Emc10 and Sp1). In the kidney, the unique GO BPs include response to ischemia (Cx3cl1, Hk2 and Panx1), blood coagulation (Comp, Hpse and Fbln1) and positive regulation of cytokine production (Postn, Panx1 and Cd274). Thus, many of the GO BPs unique to each EC phenotype, as well as those that are similar between the phenotypes, are inflammatory-related, supporting the observation that the heterogeneity of the EC proteomics contributes to cytokine-induced changes as observed in sepsis.

We further analyzed the upregulated and downregulated proteins using the PANTHER and/or GeneCards database, to identify the classes of proteins common between the three EC phenotypes. We then analyzed the log2 fold changes of 10 of the proteins that have the highest level of upregulation or downregulation compared to the control, post cytomix treatment at 4 h and 24 h. The results of this comparison for upregulated proteins at 4 h (Figure 5, panel A), 24 h (Figure 5, panel B) and the downregulated proteins at 24 h (Figure 5, panel C) are plotted. Table 1, Table 2 and Table 3 show the respective classes for each of the 10 proteins that have the highest level of upregulation or downregulation compared to the control post cytomix treatment between the three EC phenotypes upregulated at 4 h, upregulated at 24 h and downregulated at 24 h, respectively. The only common downregulated protein at 4 h was Pdcd4, a key translation factor and a regulator of apoptosis [28]. The log2 fold changes of Pdcd4 in the lung, liver and kidney were −4, −2 and −1, respectively. For the 4 h cytomix treatment, we identified upregulated proteins in the RNA metabolism, cell adhesion, defense/immunity, intercellular signaling, membrane traffic, metabolite interconversion enzyme, protein-modifying enzyme and transmembrane-signaling receptor classes. At 24 h post cytomix treatment, we identified upregulated proteins in the defense/immunity, DNA metabolism, gene-specific transcriptional regulator and anti-apoptotic classes. Unlike the upregulated proteins, none of the PANTHER classes in the downregulated proteins at 24 h were grouped in the defense/immunity class. Rather, the downregulated proteins at 24 h were grouped in the cytoskeletal class, specifically playing a role in actin binding, the extracellular matrix, inhibition of translation, the cell cycle, protein-binding activity, angiogenesis, cell proliferation, calcium binding, coagulation and metabolite interconversion enzyme classes. Several of the proteins were identified in more than one class, thus highlighting the multiple processes that are altered during the cytokine-induced changes as observed in sepsis.

The log2 fold change shown in Figure 5 indicate that, although there was an increase in the upregulated proteins across all of the EC phenotypes, the proteins were upregulated to a higher level in the liver compared to the lung and kidney at both 4 h and 24 h. This supports the observation that, while the organs, such as the liver, are more affected by inflammation starting from earlier time points, additional organs, such as the lung and kidney, are impacted in the cytokine-induced changes at later time points.

Of the 10 proteins that have the highest level of upregulation or downregulation compared to the control, the liver ECs expressed the largest number of proteins with the highest fold change, while there were still both unique and uniform differential patterns between the different EC types. For example, Vcam-1 (Vascular cell adhesion molecule 1), a critical adhesion molecule for neutrophil attachment and migration across ECs [29], was upregulated uniformly in all three EC types. Selp (P-selectin), another critical adhesion molecule regulating the leukocyte–endothelium interactions, is induced by TNF-α and IL-1β (components of cytomix) in mice [30]. Selp was found to be significantly upregulated in all three EC phenotypes as expected, but the level of its upregulation was highest in the lung, followed by the liver and the kidney. Acod1 (Aconitate decarboxylase 1) is a key regulator of immunometabolism in disease and its upregulation was found to be significantly higher in the kidney, followed by the liver and then the lung [31]. In the case of the 24 h timepoint, the lung ECs expressed Zbp1 (Z-DNA binding protein 1) to a significantly higher degree than the liver and kidney. Zbp1 regulates the host defense against pathogens by sensing viral nucleic acids [32]. Cebpb (CCAAT Enhancer Binding Protein Beta) and H2-k1 (Histocompatibility 2, K1, K region) are two proteins that play critical roles in regulating the cytokine-induced changes as observed during sepsis, and were significantly higher in the lung compared to the liver and kidney [15,33]. Rsad2 (Radical S-adenosyl methionine domain containing 2) is found to be responsive to interferon and again is found to be associated with the cytokine-induced changes as observed during sepsis [34]. Though Rsad2 was not statistically significant between the organs, its expression was found to be highest in the kidney, then the liver and lastly the lung. At 24 h, Vwf (von Willebrand factor) was significantly downregulated in the lung, compared to the liver and kidney. Vwf plays a role in maintaining hemostasis [35] and the dysfunction of the complementary and coagulation processes are well-known in the inflammatory processes as observed during sepsis [36]. Overall, these findings further indicate that during the cytokine-induced changes as observed in sepsis, many of the key proteins are differentially expressed in the ECs of different organs over time.

In order to investigate how each of the upregulated and downregulated proteins associated in each GO BP interacted with the proteins in the other BPs, gene-concept network plots (or cnetplots) were generated for the ECs in Figure 6, panels A–C. As shown in Figure 6, panel C, only four of five significant GO BPs were identified in the downregulated proteins in the liver and kidney, respectively, at 24 h. Panel A represents the 4 h upregulated cnetplot, panel B represents the 24 h upregulated cnetplot and panel C represents the 24 h downregulated cnetplot. Each cnetplot shows the interaction levels of each of the BP proteins and their shared criteria between the organs. Most importantly, the common proteins between the BPs again highlight the heterogeneity of the organ responses to the cytomix treatment and their time-dependent changes during inflammation. It is important to note that, while at the 4 h upregulated timepoint there are more unique proteins, at the 24 h upregulated timepoint, the proteins start sharing more of the BPs, indicating the commonality between the ECs as the inflammation progresses.

### 2.3. Statistical Analysis

Multivariate analysis of variance (MANOVA) was performed, using the 19 proteins that were upregulated in all three organs at both 4 h and 24 h to test the hypothesis that treatment (control vs. cytomix), organ (lung, liver, kidney) and time (4 h vs. 24 h) had a significant impact on the protein expression levels. The impact of all three factors (treatment, organ, time), as well as their corresponding interactions with the upregulated proteins were found to be statistically significant (*p* < 0.05 in all of the cases). There was only one protein that was downregulated in all three organs at both 4 h and 24 h. Analysis of variance (ANOVA) indicated that the downregulated protein was significantly impacted by both the treatment and organ (*p* < 0.05), as well as by the interaction between organ and treatment (*p* < 0.05) and interaction between organ and time (*p* < 0.05). MANOVA was used to demonstrate that 10 of the proteins that have the highest level of upregulation or downregulation compared to the control at 4 h and 24 h (Figure 5) were significantly impacted by the organ type (*p* < 0.05). ANOVA was used to identify the statistically significant differences between organs in 10 of the proteins that have the highest level of upregulation or downregulation compared to the control at 4 h and 24 h (Figure 5). This statistical analysis supports the concept that organ-specific EC proteomic expression changes significantly over time during inflammation. More information on these specific proteins can be found in the “Proteins for Statistical Analysis”, “4 h Upregulated Statistical Results”, “4 h Downregulated Statistical Results” and “24 h Downregulated Statistical Results” Excel files in the Supplementary Materials.

## 3. Discussion

In this study, we performed a global proteomic analysis to study the differential response of mouse ECs from three different organs (lung, liver and kidney) that are often most immediately affected by cytokine-induced changes as observed in sepsis [37]. The treatment of ECs with a cocktail of proinflammatory cytokines was used to induce inflammation [38]. Our results, from an analysis of more than 6000 proteins, highlighted distinct changes in the number of proteins, BPs and molecular responses distinctive for the cytokine-induced changes as observed in sepsis. The ECs from the three organs had both unique and common upregulated and downregulated proteins following treatment with the cytomix. Using global proteomic analysis allowed us to compare the time and organ-specific EC protein differences. Hence, the analysis presented here lays a proteomic framework for the better understanding of dynamic organ-specific cellular responses.

Our studies demonstrate that, during cytokine-induced changes as observed in sepsis, in addition to alterations in defense and immunity cellular responses, a number of other cellular processes, such as cell adhesion, nucleic acid metabolism, angiogenesis and apoptosis, are also impacted. More importantly, time-dependent protein expression changes were observed. While some of these responses were common in all three ECs, others were unique to one EC phenotype or another. For example, the differential expression of proteins responsible for both the induction and repression of cytokine-induced changes were organ-specific. Both the level of expression and the number of upregulated and downregulated proteins differed between the EC phenotypes, suggesting the importance of investigating organ-specific EC responses during inflammation. The bioinformatic analysis of the proteins indicated that, while the lung ECs were the most differentially expressed, there were also several, highly expressed unique proteins in both the liver and kidney ECs. These findings are consistent with our understanding that during cytokine-induced changes, a dysregulated host response to infection results in the activation of ECs, coagulation and increased cell adhesion [39,40]. Furthermore, the organ-dependent differential expression of the cellular pathways is also consistent with clinical and in vivo observations of the complexity of the inflammatory environment [41,42].

Our findings highlight a time-dependent increase in the number and magnitude of upregulated and downregulated proteins from 4 h to 24 h, with the lung ECs demonstrating the most pronounced change. Furthermore, the number of commonly shared upregulated or downregulated proteins between the EC phenotypes increased over time, as shown in the Venn diagrams. A heatmap analysis of the lung, liver and kidney biological replicates at 4 h and 24 h showed some heterogeneity of protein expression between samples within organs over time. Based on the global proteomic analysis, there are bound to be similarities between the control and experimental samples as the treatment (cytokines) is not going to change the expression level of all of the proteins. The protein expression changes occur over a much larger time frame than the gene expression. Hence, it is reasonable to expect that at 4 h, minimal differences will be observed between the treated and control samples, while the expression level increases as the treatment time progresses as evidenced by the significant increase observed at 24 h.

Interestingly, the functional enrichment analysis of the commonly upregulated proteins showed that the same top five GO BPs were observed in the ECs of all three organs, and four of these GO BPs were conserved over time, supporting the observation that similar BPs regulate the progression of inflammation over time. Furthermore, the GO BPs observed in the uniquely upregulated proteins of the ECs of different organs were also classified as inflammatory-related. While the three EC phenotypes showed similar expression for the protein-modifying enzyme and cell-adhesion classes at 4 h, at the 24 h timepoint, defense immunity and DNA metabolism had the highest organ-specific changes. These again suggest that, while the defense/immunity mechanism may be the most common response during inflammation among all three organs, other cellular mechanisms also promote the survival of the cells over time. This was also confirmed by the commonality of the nodes of interactions between each of the top five BPs upregulated for each of the EC types. The functional enrichment analysis of the downregulated proteins was different compared to the upregulated proteins, since no significant GO BPs were observed in the downregulated proteins at 4 h in the lung, liver or kidney, and only at 24 h in the liver and kidney. The identified GO BPs show how inflammation has a significant impact on blood vessel development and angiogenesis, as confirmed in other studies [43]. While Pdcd4 is the only common, downregulated protein at 4 h and all three ECs showed similar expression, at 24 h, the three ECs showed a similar expression in the calcium binding, cytoskeletal: non-motor actin binding and cell proliferation classes. These results again confirm that inflammation affects multiple cellular mechanisms of the ECs as well as the immune system and warrant further investigation.

Though this study was performed in mice ECs, our proteomic findings do contribute to our understanding of the inflammatory conditions in human ECs. The proteins, such as Selp and Vcam-1 (adhesion molecules), thrombomodulin, Prox1, Foxc2 and endothelial protein c receptor (all involved in coagulation) were evaluated in this study, with Selp, Vcam-1 and Foxc2 being upregulated in ECs. The proteome of the human ECs under inflammatory conditions exhibit responses that are similar to the findings reported in this study for mouse ECs [15,44]. For example, during the cytokine-induced changes as observed in sepsis, IL-1β (which was part of the cytomix cocktail to stimulate the mouse ECs in our study) is released and adhesion molecules (such as VCAM-1 and SELP) are upregulated from human ECs during inflammation. Furthermore, in human ECs, the proteins, such as Prox1 and Foxc2, are downregulated during inflammation which, in turn, decrease thrombomodulin and endothelial protein c receptor expression (both involved in controlling coagulation), initiating leukocyte adhesion and migration [15]. Thus, inflammatory-related pathways and BPs are dysregulated in human ECs, similar to what was observed in this study using mouse ECs.

Our findings could provide further information on the underlying mechanisms of other vascular diseases, such as atherosclerosis, and on the involvement of the mitochondria in multiple pathologies. In this proteomics study, we observed several inflammatory-related GO BPs (e.g., innate immune response, response to cytokines) that are upregulated in ECs during inflammation which are potentially impacted during atherosclerosis and may serve as targets of interest. Atherosclerosis also has a significant impact on metabolic-related processes [45,46,47,48], and we identified additional BPs that alter the reactive oxygen species, reactive nitrogen species and lipid production in ECs (e.g., the positive regulation of reactive oxygen species, metabolic process, nitric oxide biosynthetic process, response to lipid). Thus, the current study characterizes a number of underlying processes and associated proteins that occur during inflammation and encourages future proteomic studies of EC dysfunction during vascular disease progression, such as atherosclerosis. Given the roles of mitochondria in producing energy through respiration and regulating cellular metabolism, it stands to reason that mitochondrial function dysregulation may be involved in various pathologies. For example, the release of mitochondrial DNA and damage-associated molecular patterns (DAMPs) following injury, trauma or sepsis (especially within ECs) has been implicated in activating TLR9, inducing MAPK and NF-κB inflammatory pathways and disrupting the EC barrier via the targeting of HMGB1 [49]. Studies have also shown that metabolic changes (e.g., increased glucose uptake promoting tumor growth) in malignant cells help to compensate for malfunctions in the respiratory chain, and it has been hypothesized that the blockage of glycolysis could favor oxidative phosphorylation and an anti-metastatic phenotype [50]. From our proteomic analysis, we have identified several mitochondrial-related BPs and associated proteins therein that contribute to the inflammatory and metabolic dysregulation in ECs [45].

In summary, we used a cytokine cocktail comprising of TNF-α, IL-1β and IFN-γ to treat different organ-specific ECs for 4 h or 24 h and modelled the cytokine-induced changes as observed in sepsis. Our study demonstrates that during the inflammatory process, the organ-specific EC proteomic expression changes over time, and there are unique EC-specific GO BPs and proteins that are differentially expressed. Post-translational modification of proteins may also play an important role in regulating the EC function and should be investigated in future studies. Finally, it will be important to build a global cellular pathway map to investigate the underlying mechanisms of proteomic changes in ECs and their downstream phenotypic effects on cell functions, such as leukocyte–endothelial interaction and permeability. These effects are often observed during cytokine-induced changes and may play important roles in organ dysfunction and failure [36,51]. Though our initial goal was to explore and examine the entire proteome in ECs, our long-term goal is to identify organ-specific EC proteins that could serve as therapeutic targets for precision medicine. As we identify EC-specific proteins that can be used to phenotype patients, we will confirm and validate these specific proteins experimentally (e.g., using Western Blotting).

## 4. Materials and Methods

### 4.1. Endothelial Cell Preparation

Mouse Primary Liver Sinusoidal ECs (cat# C57-6017), C57BL/6 Mouse Primary Lung Microvascular ECs (cat# C57-6011) and C57BL/6 Mouse Primary Kidney ECs (cat# C57-6014) were purchased from Cell Biologics (Chicago, IL, USA). The ECs were isolated from C57BL/6 pathogen-free laboratory mice and were negative for bacteria, yeast, fungi and mycoplasma. The ECs were grown on gelatin-coated T25 flasks at 37 °C in 5% CO_2_, according to the manufacturer’s specifications for each type of mouse EC. Each EC type was used in experiments at passages 4–5. The ECs were harvested and plated in 6 well plates at a cell concentration of 0.25 × 10^6^ cells/well and grown for 3–4 days until the cells formed a confluent monolayer. The cells (four wells/condition) were treated with buffer (control) or Cytomix (TNF-α (10 ng/mL, R&D, 410-MT-010), IL-1β (5 ng/mL, R&D, 401-ML-005), IFN-γ (100 ng/mL, 485-MI-100)) for 4 h or 24 h [23,24,25,26]. This well-described cocktail of clinically relevant inflammatory cytokines has been used to model the inflammatory milieu of sepsis and has been used extensively to model cytokine-induced cellular changes during septic conditions in vitro, using epithelial and ECs [23,24,25,26,38,52]. The cells were harvested by treatment with 0.25% Trypsin-EDTA Solution (Cell Biologics, cat# 6914) and cell scrapping. The collected cells were centrifuged, and cell pellets stored at −70 °C before proteomic analysis.

### 4.2. Global Label-Free Proteomic Analysis

For the label-free global proteomics studies, the proteins were extracted by adding 6M guanidium hydrochloride buffer and dilution buffer (25 mM Tris, 10% acetonitrile). The proteins were digested with Lys-C for 4 h at 37 °C. A second digestion was achieved by overnight incubation with trypsin. The incubated solution was acidified and centrifuged at 4500× *g* for 5 min. The supernatants consisting of peptides were loaded onto activated in-house constructed cation stage tips [53]. The peptides from each sample were eluted into six fractions using elution buffers, as previously described [54]. Mass spectrometry (MS) analyses were performed on these fractions, using the Quadrupole Orbitrap Mass Spectrometer (Q Exactive—ThermoFisher Scientific, Waltham, MA, USA) [55]. The desalted tryptic peptide samples were loaded onto an Acclaim PepMap 100 pre-column (75 μm × 2 cm, ThermoFisher Scientific) and separated by Easy-Spray PepMap RSLC C18 column with an emitter (2 μm particle size, 15 cm × 50 μm ID, ThermoFisher Scientific) by an Easy nLC system with Easy Spray Source (ThermoFisher Scientific). To elute the peptides, a mobile-phase gradient was run using an increasing concentration of acetonitrile. The peptides were loaded in buffer A (0.1% (*v/v*) formic acid) and eluted with a nonlinear 145-min gradient as follows: 0–25% buffer B (15% (*v/v*) of 0.1% formic acid and 85% (*v/v*) of acetonitrile) for 80 min, 25–40% B for 20 min, 40–60% B for 20 min and 60–100% B for 10 min. The column was then washed with 100% buffer B for 5 min and re-equilibrated, 50% buffer B for 5 min and re-equilibrated with buffer A for 5 min. The flow rate was maintained at 300 nl/min. Electron spray ionization was delivered at a spray voltage of −1500 V. The MS/MS fragmentation was performed on the five most abundant ions in each spectrum using collision-induced dissociation with dynamic exclusion (excluded for 10.0 s after one spectrum), with automatic switching between the MS and MS/MS modes. The complete system was entirely controlled by Xcalibur software. Mass spectra processing was performed with Proteome Discoverer version 2.5. The generated de-isotoped peak list was submitted to an in-house Mascot server 2.2.07 for searching against the Swiss-Prot database (Release 2013_01, version 56.6, 538,849 sequences) and Sequest HT database. Both Mascot and Sequest search parameters were set as follows: species, mus musculus; enzyme, trypsin with maximal two missed cleavage; fixed modification, cysteine carboxymethylation; 10 ppm mass tolerance for precursor peptide ions; 0.02 Da tolerance for MS/MS fragment ions. For dynamic modifications, we used oxidation/+15.995 Da (M) and N-terminal modification Met-loss/-131.040 Da (M); additional information on the alkylation/reduction can be found elsewhere [56]. The proteomics of the ECs were investigated using a factorial design of three factors: treatment (control vs. cytomix); organ (lung, liver, kidney) and time (4 h vs. 24 h) with *n* = 3 per condition for a total of 36 samples. The mass spectrometry proteomics data were deposited to the ProteomeXchange Consortium via the PRIDE [57,58] partner repository with the dataset identifier PXD031804 and 10.6019/PXD031804. The reviewer account details are username: “reviewer_pxd031804@ebi.ac.uk”, password: “3IUdGW3V”.

### 4.3. Bioinformatic Analysis

The protein lists generated by the Proteome Discoverer (v. 2.5) were uploaded into the R programming language for Differentially Expressed Protein (DEP) identification [59]. Within R, the Bioconductor software was used to access the packages used for analysis and visualization [60,61]. The proteins with a log2 fold change > 1 and *p* < 0.05 were considered to qualify as upregulated. Similarly, any protein with a log2 fold change < −1 and *p* < 0.05 were considered downregulated with target false discovery rate (FDR) set to 5%. These protein lists were then separated and split into smaller lists for each. Figure S3 (Supplementary Materials) shows a flow chart of the process used for bioinformatic analysis. The upregulated and downregulated proteins were compiled for each dataset and then compared to one another across the timepoints, using volcano plots. In addition, the upregulated and downregulated ECs were all compared to one another in Venn diagrams at the two different timepoints to elucidate the commonality and difference between each of the categories using the venn.diagram function, as part of the Venn diagram package in R [62]. From these plots, we were able to compile the lists of proteins specific to the EC type and those that were common across timepoints and organs. Heatmaps of normalized protein expression for the sample replicates for each organ, treatment and time factor were created from the data; hierarchical sample clustering by complete linkage method using Euclidean distance was implemented and the data were scaled before clustering. White bars in the heatmaps represent proteins that were not expressed. For normalization, the Proteome Discoverer software uses total peptide amounts to sum the peptide group abundances for each sample and determines the maximum sum for all of the files. The normalization factor is the factor of the sum of the sample and the maximum sum in all of the files. In order to identify the Biological Processes (BPs) most affected, we performed a Gene Ontology (GO) Over Representation Analysis (ORA) across each list of upregulated DEPs [63]. GO Biological Processes (BPs) were considered statistically significant if *p* < 0.05. These analyses were completed using the R Package clusterProfiler [64]. ORA accepts a list of proteins to identify, in which categories from the BP ontology are overrepresented [65]. Dotplots for each EC type at 4 h and 24 h, evaluating significant BPs that were common across organs, were generated using the compareCluster function, as part of the clusterprofiler package [64,66]. Once the dotplots were produced, gene-concept network plots (or cnetplots) among the different EC types at 4 h and 24 h were created, using the compareCluster output and cnetplot function. Finally, the PANTHER or GeneCards database was used to identify the distinct classes that the common upregulated and downregulated proteins between the lung, liver and kidney ECs at 4 h and 24 h were in, and bar plots illustrating the log2 fold change expression amongst these common proteins were created in R.

### 4.4. Statistical Analysis

The normalized abundance values for the proteins that were detected in at least two of the three replicates in both of the treatments, at both time points and in all three organs (19 upregulated proteins and 1 downregulated protein) were used for the statistical analysis [67,68]. The impact of treatment (control vs. cytomix), time (4 h vs. 24 h) and organ (lung, liver, kidney) on the proteomic expression of ECs was investigated using multivariate analysis of variance (MANOVA) for the 19 upregulated proteins and analysis of variance (ANOVA) for the one downregulated protein in JMP Pro (version 16.1). MANOVA was used to investigate the impact of organ specificity on fold-change expression between the three EC types. Additionally, ANOVA was used to analyze statistically significant differences between the organs in 10 of the proteins that had the highest level of upregulation or downregulation compared to the control post cytomix treatment at 4 h and 24 h. Values of *p* < 0.05 were considered statistically significant.

## Figures and Tables

**Table 1:** The PANTHER/GeneCards classes of 10 of the proteins that have the highest level of upregulation compared to control at 4 h post cytomix treatment. As shown, most proteins are categorized within the defense/immunity, cell adhesion and metabolite interconversion enzyme classes and belong to multiple classes.

Protein Class	Protein(s)
RNA Metabolism	Zfp36
Cell Adhesion	Vcam-1, Selp
Defense/Immunity	Selp, Ccl2, Isg15, Tlr2, Acod1
Intercellular Signaling	Ccl2
Membrane Traffic	Tnfaip2
Metabolite Interconversion Enzyme	Ptgs2, Acod1
Protein Modifying Enzyme	Ripk2
Transmembrane Signal Receptor	Tlr2
Cell-cell signaling	Isg15

**Table 2:** The PANTHER/GeneCards classes of 10 of the proteins that have the highest level of upregulation compared to control at 24 h post cytomix treatment. As shown, most proteins are categorized within the defense/immunity class, followed by the protein modifying enzyme class.

Protein Class	Protein(s)
DNA Metabolism	Zbp1
Defense/Immunity	Ifit2, Ifit1, H2-k1, Ifi44, Rsad2, Zbp1, Cebpb
Gene Specific Transcriptional Regulator	Cebpb
Protein Modifying Enzyme	Irak2, Ripk2
Anti-apoptosis	Parp14

**Table 3:** The PANTHER/GeneCards classes of 10 of the proteins that have the highest level of downregulation compared to control at 24 h post cytomix treatment. As shown, most proteins are categorized within the protein binding activity, extracellular matrix and cell cycle classes and belong to multiple classes.

Protein Class	Protein(s)
Cytoskeletal: Non-Motor Actin Binding	Fli1
Extracellular Matrix	Vwf, Hyal2
Coagulation	Vwf
Translation Inhibitor (apoptosis)	Pdcd4
Cell Cycle	Ptprb, Rgcc
Protein Binding Activity	She, Cbr4, Ednrb
Angiogenesis	Tie1
Cell Proliferation	Hyal2
Metabolite Interconversion Enzyme	Cbr4
Calcium Binding	Ednrb

## Data Availability

The mass spectrometry proteomics data were deposited to the ProteomeXchange Consortium via the PRIDE [57,58] partner repository with the dataset identifier PXD031804 and 10.6019/PXD031804. The reviewer account details are username: “reviewer_pxd031804@ebi.ac.uk”, password: “3IUdGW3V”.

## Supplementary Materials

The following supporting information can be downloaded at: www.mdpi.com/article/10.3390/ijms23158399/s1.

## Abbreviations

ANOVA	Analysis of Variance
Acod1	Aconitate decarboxylase 1
Arg1	Arginase 1
BP	Biological process
Bcl11b	B cell leukemia/lymphoma 11B
Crp	C-reactive protein
Ctla2a	Cytotoxic T lymphocyte-associated protein 2 alpha
C1qc	Complement component 1, q subcomponent, C chain
Clec2d	C-type lectin domain family 2, member d
Cebpb	CCAAT/enhancer binding protein (C/EBP), beta
Cd44	Cluster of differentiation 44
Cx3cl1	Chemokine (C-X3-C motif) ligand 1
Comp	Cartilage oligomeric matrix protein
Cd274	Cluster of differentiation 274
Ccl2	C-C motif chemokine ligand 2
Cbr4	Carbonyl reductase 4
DAMPs	Damage-associated molecular patterns
DEP	Differentially expressed protein
EC	Endothelial cell
Ets1	ETS proto-oncogene 1
Ednrb	Endothelin receptor type b
Emc10	ER membrane protein complex subunit 10
Fam181b	Family with sequence similarity 181, member B
Fbln1	Fibulin 1
Fli1	Friend Leukemia Integration 1
Foxc2	Forkhead box c2
GO	Gene ontology
Gimap3	GTPase of immunity associated protein 3
Gimap5	GTPase of immunity associated protein 5
Gja1	Gap junction protein, alpha 1
Hk2	Hexokinase 2
Hpse	Heparinase
H2-k1	Histocompatibility 2, K1, K region
Hyal2	Hyaluronidase 2
Hmgb1	High mobility group box 1
IL-1β	Interleukin-1 beta
IFN-γ	Interferon-gamma
Il6	Interleukin-6
Ifit2	Interferon-induced protein with tetratricopeptide repeats 2
Ifit1	Interferon-induced protein with tetratricopeptide repeats 1
Ifi44	Interferon-induced protein with tetratricopeptide repeats 44
Irak2	Interleukin 1 receptor associated kinase 2
Irf1	Interferon regulatory factor 1
Lys-C	Lysyl-endopeptidase
MANOVA	Multivariate analysis of variance
MS/MS	Tandem mass spectrometry
Met	Methionine
(m)M	Millimolar
Mospd3	Motile sperm domain containing 3
Mapk	Mitogen-activated protein kinase
NF-kβ	Nuclear factor kappa beta
Nrp1	Neuropilin 1
Nsd2	Nuclear receptor-binding SET domain protein 2
ORA	Overrepresentation analysis
Parp14	Poly(ADP-Ribose) polymerase family member 14
Panx1	Pannexin 1
Ptprb	Protein tyrosine phosphatase receptor type B
Ptgs2	Prostaglandin-endoperoxide synthase 2
Postn	Periostin
Ptpn5	Protein tyrosine phosphatase, non-receptor type 5
Pdcd4	Programmed cell death 4
PRIDE	Proteomics identification database
Prox1	Prospero homeobox 1
Rsad2	Radical S-adenosyl methionine domain containing 2
Rgcc	Regulator of cell cycle
Ripk2	Receptor interacting serine/threonine kinase 2
RSLC	Rapid separation liquid chromatography
Selp	P-selectin
Sox13	SRY (sex determining region Y)-box 13
Smim4	Small integral membrane protein 4
Sp1	Sp1 transcription factor
She	Src homolog 2 domain containing E
TNF-α	Tumor necrosis factor-alpha
Timm17a	Translocase of inner mitochondrial membrane 17a
Tlr4	Toll-like receptor 4
Trex1	Three prime repair exonuclease 1
Tap1	Transporter 1, ATP binding cassette subfamily B member
Tlr9	Toll-like receptor 9
Tie1	Tyrosine kinase with immunoglobulin-like and egf-like domains 1
Tnfaip2	Tumor necrosis factor alpha-induced protein 2
Vcam-1	Vascular cell adhesion molecule 1
Vwf	von Willebrand factor

## References

[1] Yang Q., Wijerathne H., Langston J., Kiani M., Kilpatrick L. (2021). Emerging Approaches to Understanding Microvascular Endothelial Heterogeneity: A Roadmap for Developing Anti-Inflammatory Therapeutics. Int. J. Mol. Sci..

[2] Wijerathne H., Langston J.C., Yang Q., Sun S., Miyamoto C., Kilpatrick L.E., Kiani M.F. (2021). Mechanisms of radiation-induced endothelium damage: Emerging models and technologies. Radiother. Oncol..

[3] Maniatis N.A., Orfanos S.E. (2008). The endothelium in acute lung injury/acute respiratory distress syndrome. Curr. Opin. Crit. Care.

[4] Nourshargh S., Alon R. (2014). Leukocyte migration into inflamed tissues. Immunity.

[5] Kiseleva R., Glassman P., Greineder C., Hood E., Shuvaev V., Muzykantov V. (2018). Targeting therapeutics to endothelium: Are we there yet?. Drug Deliv. Transl. Res..

[6] Augustin H.G., Koh G.Y. (2017). Organotypic vasculature: From descriptive heterogeneity to functional pathophysiology. Science.

[7] Aird W.C. (2012). Endothelial Cell Heterogeneity. Cold Spring Harb. Perspect. Med..

[8] Marcu R., Choi Y.J., Xue J., Fortin C.L., Wang Y., Nagao R.J., Xu J., MacDonald J.W., Bammler T.K., Murry C.E. (2018). Human Organ-Specific Endothelial Cell Heterogeneity. iScience.

[9] Rhee C., Dantes R., Epstein L., Murphy D.J., Seymour C.W., Iwashyna T.J., Kadri S.S., Angus D.C., Danner R.L., Fiore A.E. (2017). Incidence and Trends of Sepsis in US Hospitals Using Clinical vs Claims Data, 2009–2014. J. Am. Med. Assoc..

[10] Angus D.C., Linde-Zwirble W.T., Lidicker J., Clermont G., Carcillo J., Pinsky M.R. (2001). Epidemiology of severe sepsis in the United States: Analysis of incidence, outcome, and associated costs of care. Crit. Care Med..

[11] Iskander K.N., Osuchowski M.F., Stearns-Kurosawa D.J., Kurosawa S., Stepien D., Valentine C., Remick D.G. (2013). Sepsis: Multiple Abnormalities, Heterogeneous Responses, and Evolving Understanding. Physiol. Rev..

[12] Hattori Y., Hattori K., Suzuki T., Matsuda N. (2017). Recent advances in the pathophysiology and molecular basis of sepsis-associated organ dysfunction: Novel therapeutic implications and challenges. Pharmacol. Ther..

[13] Raju S.M., Jahnavi V., Kamaraju R.S., Sritharan V., Rajkumar K., Natarajan S., Kumar A.D., Burgula S. (2016). Continuous evaluation of changes in the serum proteome from early to late stages of sepsis caused by Klebsiella pneumoniae. Mol. Med. Rep..

[14] Hasin Y., Seldin M., Lusis A. (2017). Multi-omics approaches to disease. Genome Biol..

[15] Langston J.C., Rossi M.T., Yang Q., Ohley W., Perez E., Kilpatrick L.E., Prabhakarpandian B., Kiani M.F. (2022). Omics of Endothelial Cell Dysfunction in Sepsis. Vasc. Biol..

[16] Pimienta G., Heithoff D.M., Rosa-Campos A., Tran M., Esko J.D., Mahan M.J., Marth J.D., Smith J.W. (2019). Plasma Proteome Signature of Sepsis: A Functionally Connected Protein Network. Proteomics.

[17] Toledo A.G., Golden G., Campos A.R., Cuello H., Sorrentino J., Lewis N., Varki N., Nizet V., Smith J.W., Esko J.D. (2019). Proteomic atlas of organ vasculopathies triggered by Staphylococcus aureus sepsis. Nat. Commun..

[18] Luo T., Yan H.P., Li X., Deng Y.C., Huang J.T., Li L.P., Xiao Z.H., Lu X.L. (2021). Proteomic analysis identified potential age-associated prognostic biomarkers in pneumonia-derived paediatric sepsis. Proteom. Clin. Appl..

[19] Jiao J., Gao M., Zhang H.L., Wang N., Xiao Z.H., Liu K., Yang M.S., Wang K.K., Xiao X.Z. (2014). Identification of potential biomarkers by serum proteomics analysis in rats with sepsis. Shock.

[20] Scicluna B.P., van Vught L.A., Zwinderman A.H., Wiewel M.A., Davenport E.E., Burnham K.L., Nurnberg P., Schultz M.J., Horn J., Cremer O.L. (2017). Classification of patients with sepsis according to blood genomic endotype: A prospective cohort study. Lancet Respir. Med..

[21] Davenport E.E., Burnham K.L., Radhakrishnan J., Humburg P., Hutton P., Mills T.C., Rautanen A., Gordon A.C., Garrard C., Hill A.V.S. (2016). Genomic landscape of the individual host response and outcomes in sepsis: A prospective cohort study. Lancet Respir. Med..

[22] Wong H.R., Cvijanovich N., Lin R., Allen G.L., Thomas N.J., Willson D.F., Freishtat R.J., Anas N., Meyer K., Checchia P.A. (2009). Identification of pediatric septic shock subclasses based on genome-wide expression profiling. BMC Med..

[23] Wang L.F., Chung J., Gill S.E., Mehta S. (2019). Quantification of adherens junction disruption and contiguous paracellular protein leak in human lung endothelial cells under septic conditions. Microcirculation.

[24] Wong E., Nguyen N., Hellman J. (2021). Isolation of Primary Mouse Lung Endothelial Cells. J. Vis. Exp..

[25] Liu S.G., Stolz D.B., Sappington P.L., Macias C.A., Killeen M.E., Tenhunen J.J., Delude R.L., Fink M.P. (2006). HMGB1 is secreted by immunostimulated enterocytes and contributes to cytomix-induced hyperpermeability of Caco-2 monolayers. Am. J. Physiol. Cell Physiol..

[26] Julian M.W., Bao S.Y., Knoell D.L., Fahy R.J., Shao G.H., Crouser E.D. (2011). Intestinal epithelium is more susceptible to cytopathic injury and altered permeability than the lung epithelium in the context of acute sepsis. Int. J. Exp. Pathol..

[27] Ashburner M., Ball C.A., Blake J.A., Botstein D., Butler H., Cherry J.M., Davis A.P., Dolinski K., Dwight S.S., Eppig J.T. (2000). Gene Ontology: Tool for the unification of biology. Nat. Genet..

[28] Jiang Y., Jia Y.F., Zhang L.N. (2017). Role of programmed cell death 4 in diseases: A double-edged sword. Cell. Mol. Immunol..

[29] Kilpatrick L.E., Kiani M.F. (2020). Experimental Approaches to Evaluate Leukocyte-Endothelial Cell Interactions in Sepsis and Inflammation. Shock.

[30] McEver R.P. (2015). Selectins: Initiators of leucocyte adhesion and signalling at the vascular wall. Cardiovasc. Res..

[31] Wu R.L., Chen F., Wang N., Tang D.L., Kang R. (2020). ACOD1 in immunometabolism and disease. Cell. Mol. Immunol..

[32] Jiao H.P., Wachsmuth L., Kumari S., Schwarzer R., Lin J., Eren R.O., Fisher A., Lane R., Young G.R., Kassiotis G. (2020). Z-nucleic-acid sensing triggers ZBP1-dependent necroptosis and inflammation. Nature.

[33] Xu C., Xu J.B., Lu L., Tian W.D., Ma J.L., Wu M. (2020). Identification of key genes and novel immune infiltration-associated biomarkers of sepsis. Innate Immun..

[34] Reyes M., Filbin M.R., Bhattacharyya R.P., Sonny A., Mehta A., Billman K., Kays K.R., Pinilla-Vera M., Benson M.E., Cosimi L.A. (2021). Plasma from patients with bacterial sepsis or severe COVID-19 induces suppressive myeloid cell production from hematopoietic progenitors in vitro. Sci. Transl. Med..

[35] Drakeford C., O’Donnell J.S. (2017). Targeting von Willebrand Factor-Mediated Inflammation. Arterioscler. Thromb. Vasc. Biol..

[36] Simmons J., Pittet J.F. (2015). The coagulopathy of acute sepsis. Curr. Opin. Anesthesiol..

[37] Caraballo C., Jaimes F. (2019). Organ Dysfunction in Sepsis: An Ominous Trajectory From Infection To Death. Yale J. Biol. Med..

[38] Wang L., Mehta S., Brock M., Gill S.E. (2017). Inhibition of Murine Pulmonary Microvascular Endothelial Cell Apoptosis Promotes Recovery of Barrier Function under Septic Conditions. Mediat. Inflamm..

[39] Dolmatova E.V., Wang K.K., Mandavilli R., Griendling K.K. (2021). The effects of sepsis on endothelium and clinical implications. Cardiovasc. Res..

[40] Joffre J., Hellman J., Ince C., Ait-Oufella H. (2020). Endothelial Responses in Sepsis. Am. J. Respir. Crit. Care Med..

[41] Nedeva C., Menassa J., Puthalakath H. (2019). Sepsis: Inflammation Is a Necessary Evil. Front. Cell Dev. Biol..

[42] Seymour C.W., Kennedy J.N., Wang S., Chang C.C.H., Elliott C.F., Xu Z.Y., Berry S., Clermont G., Cooper G., Gomez H. (2019). Derivation, Validation, and Potential Treatment Implications of Novel Clinical Phenotypes for Sepsis. J. Am. Med. Assoc..

[43] Jeong J.H., Ojha U., Lee Y.M. (2021). Pathological angiogenesis and inflammation in tissues. Arch. Pharmacal Res..

[44] Lupu F., Kinasewitz G., Dormer K. (2020). The role of endothelial shear stress on haemodynamics, inflammation, coagulation and glycocalyx during sepsis. J. Cell. Mol. Med..

[45] Malekmohammad K., Bezsonov E.E., Rafieian-Kopaei M. (2021). Role of Lipid Accumulation and Inflammation in Atherosclerosis: Focus on Molecular and Cellular Mechanisms. Front. Cardiovasc. Med..

[46] Nitz K., Lacy M., Atzler D. (2019). Amino Acids and Their Metabolism in Atherosclerosis. Arterioscler. Thromb. Vasc. Biol..

[47] Wang X.J., Luo D., Wu S.S. (2021). Molecular Dysfunctions of Mitochondria-Associated Endoplasmic Reticulum Contacts in Atherosclerosis. Oxidative Med. Cell. Longev..

[48] Xu J., Kitada M., Ogura Y., Koya D. (2021). Relationship Between Autophagy and Metabolic Syndrome Characteristics in the Pathogenesis of Atherosclerosis. Front. Cell Dev. Biol..

[49] Itagaki K., Rica I., Konecna B., Kim H.I., Park J., Kaczmarek E., Hauser C.J. (2021). Role of Mitochondria-Derived Danger Signals Released After Injury in Systemic Inflammation and Sepsis. Antioxid. Redox Signal..

[50] Zhunina O.A., Yabbarov N.G., Grechko A.V., Starodubova A.V., Ivanova E., Nikiforov N.G., Orekhov A.N. (2021). The Role of Mitochondrial Dysfunction in Vascular Disease, Tumorigenesis, and Diabetes. Front. Mol. Biosci..

[51] Hotchkiss R.S., Moldawer L.L., Opal S.M., Reinhart K., Turnbull I.R., Vincent J.L. (2016). Sepsis and septic shock. Nat. Rev. Dis. Primers.

[52] Wang L.F., Taneja R., Wang W., Yao L.J., Veldhuizen R.A.W., Gill S.E., Fortin D., Inculet R., Malthaner R., Mehta S. (2013). Human Alveolar Epithelial Cells Attenuate Pulmonary Microvascular Endothelial Cell Permeability under Septic Conditions. PLoS ONE.

[53] Molina-Franky J., Plaza D.F., Merali C., Merali S., Barrero C., Arevalo-Pinzon G., Patarroyo M.E., Patarroyo M.A. (2021). A novel platform for peptide-mediated affinity capture and LC-MS/MS identification of host receptors involved in Plasmodium invasion. J. Proteom..

[54] Kulak N.A., Pichler G., Paron I., Nagaraj N., Mann M. (2014). Minimal, encapsulated proteomic-sample processing applied to copy-number estimation in eukaryotic cells. Nat. Methods.

[55] Scheltema R.A., Hauschild J.P., Lange O., Hornburg D., Denisov E., Damoc E., Kuehn A., Makarov A., Mann M. (2014). The Q Exactive HF, a Benchtop Mass Spectrometer with a Pre-filter, High-performance Quadrupole and an Ultra-high-field Orbitrap Analyzer. Mol. Cell. Proteom..

[56] Quinn C., Rico M.C., Merali C., Merali S. (2022). Dysregulation of S-adenosylmethionine Metabolism in Nonalcoholic Steatohepatitis Leads to Polyamine Flux and Oxidative Stress. Int. J. Mol. Sci..

[57] Perez-Riverol Y., Bai J.W., Bandla C., Garcia-Seisdedos D., Hewapathirana S., Kamatchinathan S., Kundu D.J., Prakash A., Frericks-Zipper A., Eisenacher M. (2022). The PRIDE database resources in 2022: A hub for mass spectrometry-based proteomics evidences. Nucleic Acids Res..

[58] Deutsch E.W., Bandeira N., Sharma V., Perez-Riverol Y., Carver J.J., Kundu D.J., Garcia-Seisdedos D., Jarnuczak A.F., Hewapathirana S., Pullman B.S. (2020). The ProteomeXchange consortium in 2020: Enabling “big data” approaches in proteomics. Nucleic Acids Res..

[59] Lee M.H. (2021). Data analysis with RStudio: An easygoing introduction. Biometrics.

[60] Morgan M. BiocManager: Access the Bioconductor Project Package Repository. https://CRAN.R-project.org/package=BiocManager.

[61] Gentleman R., Carey V., Bates D., Bolstad B., Dettling M., Dudoit S., Ellis B., Gautier L., Ge Y., Gentry J. (2004). Bioconductor: Open software development for computational biology and bioinformatics. Genome Biol..

[62] Chen H. VennDiagram: Generate High-Resolution Venn and Euler Plots. Ttps://CRAN.R-project.org/package=VennDiagram.

[63] Carbon S., Douglass E., Dunn N., Good B., Harris N.L., Lewis S.E., Mungall C.J., Basu S., Chisholm R.L., Dodson R.J. (2019). The Gene Ontology Resource: 20 years and still Going strong. Nucleic Acids Res..

[64] Wu T., Hu E., Xu S., Chen M., Guo P., Dai Z., Feng T., Zhou L., Tang W., Zhan L. (2021). clusterProfiler 4.0: A universal enrichment tool for interpreting omics data. Innovation.

[65] Boyle E., Weng S., Gollub J., Jin H., Botstein D., Cherry J., Sherlock G. (2004). GO::TermFinder—Open source software for accessing Gene Ontology information and finding significantly enriched Gene Ontology terms associated with a list of genes. Bioinformatics.

[66] Yu G.C., Wang L.G., Han Y.Y., He Q.Y. (2012). ClusterProfiler: An R Package for Comparing Biological Themes Among Gene Clusters. Omics A J. Integr. Biol..

[67] Zhang X.F., Smits A.H., van Tilburg G.B.A., Ovaa H., Huber W., Vermeulen M. (2018). Proteome-wide identification of ubiquitin interactions using UbIA-MS. Nat. Protoc..

[68] Lin T. Proteomics Data Analysis (2/3): Data Filtering and Missing Value Imputation. https://datascienceplus.com/proteomics-data-analysis-2-3-data-filtering-and-missing-value-imputation/.

