# Supplementary material for: Molecular Framework of Mouse Endothelial Cell Dysfunction during Inflammation: A Proteomics Approach
Source: Int J Mol Sci. 2022 Jul 29;23(15):8399. doi: 10.3390/ijms23158399 (PMC9369400; doi:10.3390/ijms23158399)
Supplement: Supplementary file 1 [file ijms-23-08399-s001.zip › Supplement Figures.pdf]

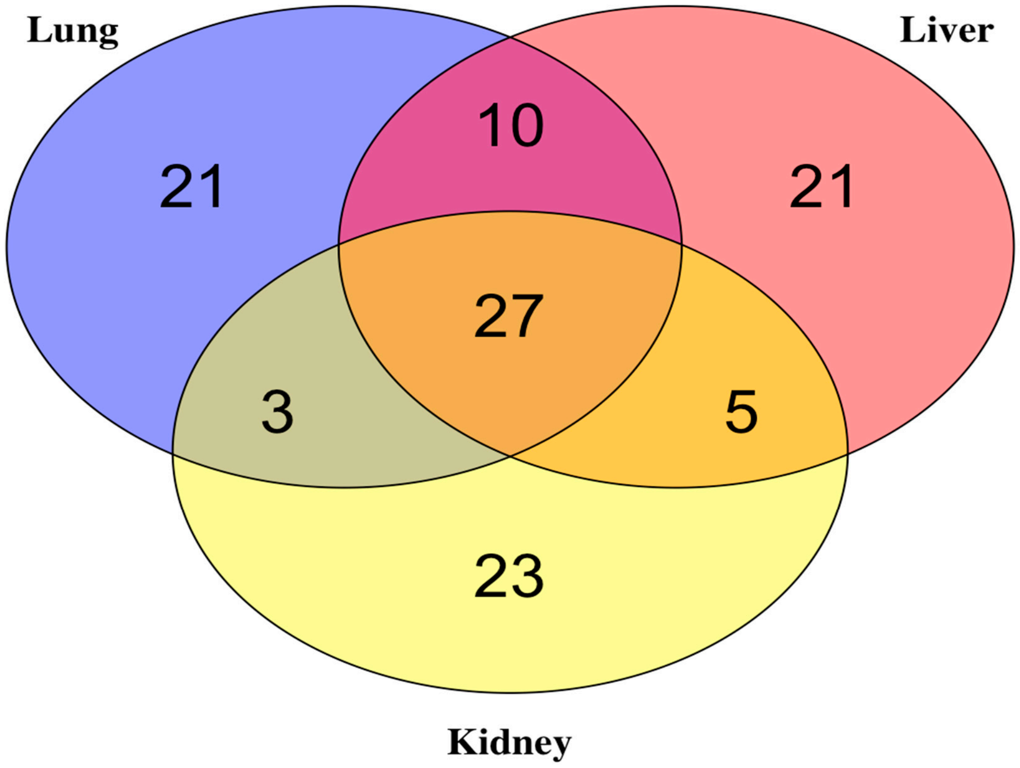

Figure S1: Venn diagram of common and unique proteins upregulated across the lung, liver and kidney at 4 hours based on the GO BP.

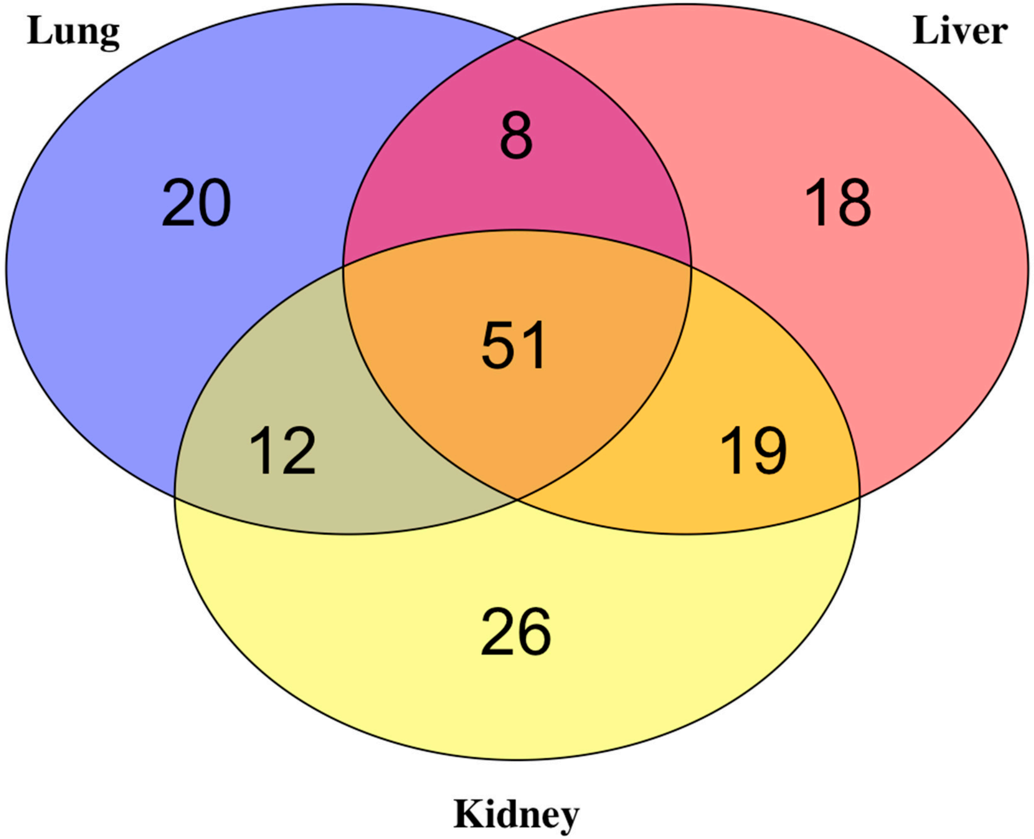

Figure S2: Venn diagram of common and unique proteins upregulated across the lung, liver and kidney at 24 hours based on the GO BP.

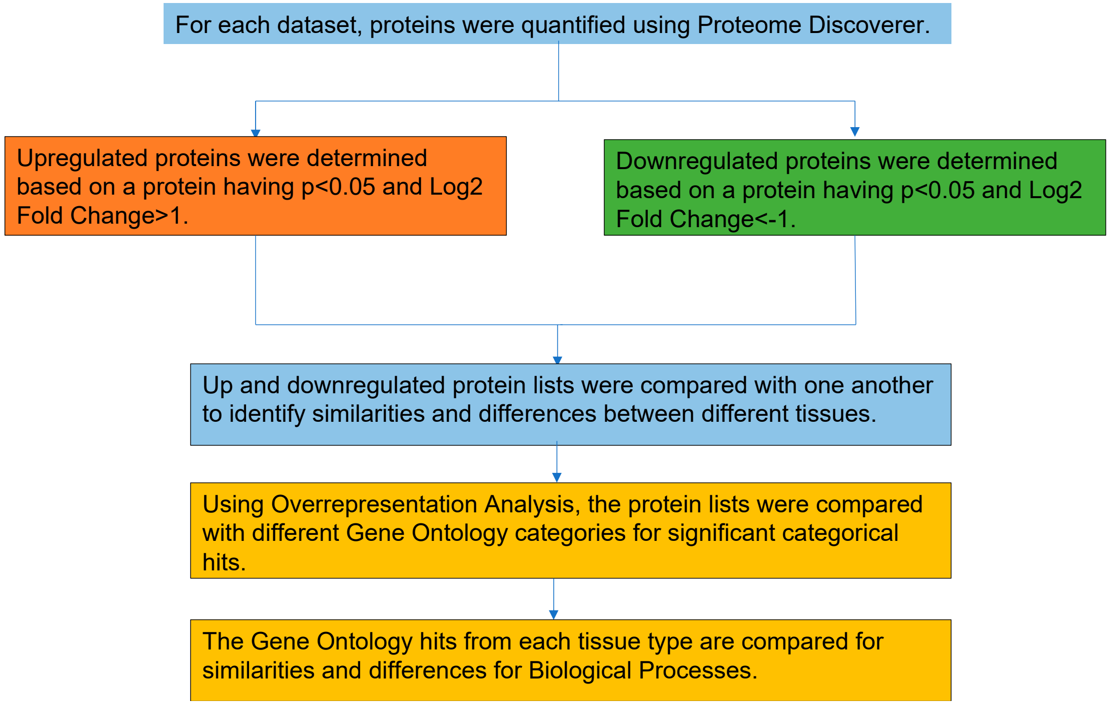

Figure S3: Process flow diagram for proteomic analysis of the ECs.
